# A case of stings in humans caused by *Sclerodermus* sp. in Italy

**DOI:** 10.1186/1678-9199-20-11

**Published:** 2014-03-31

**Authors:** Roberto Amerigo Papini

**Affiliations:** 1 Dipartimento di Scienze Veterinarie, Viale delle Piagge 2, Pisa 56124, Italy

**Keywords:** *Sclerodermus*, Bethylid wasps, Stings, Italy

## Abstract

In the last years, stings of *Sclerodermus* species in humans have been sporadically reported in Italy. In order to draw attention to these bethylid wasps of medical importance, we report the case of documented *Sclerodermus* sp. stings on the dorsum, abdomen, arms, and thighs of a 40-year-old man and his wife. The sting sites developed raised red itchy rash. The source of environmental contamination was identified in a worm-eaten sofa purchased from a used furniture dealer and placed in the living room about a month and half earlier. The lesions on the man and his wife rapidly healed within 3 to 4 days once they left the house and treatment for the lesions was instituted. Physicians, dermatologists, medical and public health entomologists, as well as specific categories of workers should be aware of the risk of exposure to *Sclerodermus* stings.

## Background

*Sclerodermus domesticus* (Latreille 1809) is an aculeate insect of the family Bethylidae, order Hymenoptera [1-11]. It is also called *Scleroderma domesticum*, *Scleroderma domestica* or *Sclerodermae domesticae*[7,10]. This species is antlike in appearance, brownish black in color, and 2–4 mm in length [4,6,9]. Females are apterous whereas males are winged, die shortly after mating, and are rarely found [1,2,7]. This bethylid wasp is parasitoid and needs xylophagous larvae of Coleoptera or Lepidoptera as hosts to complete its life cycle [10]. Larvae of Anobiidae (Coleoptera), common household pests, may serve as hosts for *S. domesticus*. These include *Anobium striatum*, *Hylotrupes bajulus*, *Lasioderma serricorne*, *Nicobium castaneum* and *Oligomerus brunneus*[1-3,5-7].

Adult anobiid powderpost beetles lay eggs on a wide variety of wood, including furniture (chairs, beds, wardrobes, sofas) and house structures (beams, door and window frames, sills, wall panels). As soon as Anobiidae larvae hatch, they feed on the wood of furniture and house structures where they grow and dig galleries, earning them the name “woodworm” or “wood borer” [1,11]. When *S. domesticus* females are ready to lay eggs, they search for host larvae entering into holes dug in the wood. Their abdominal stinger, which evolved from the ovipositor, is localized on the rear of the body and communicates with a venom gland, like in other Hymenoptera. Once the meeting takes place, *S. domesticus* females fight for some days against the host larva inflicting many stings. In doing so, *S. domesticus* females inject their venom into prey which affects the larval motor neurons and paralyze the host. They then feed on the larval hemolymph and lay 30–60 eggs on the host’s dorsal surface. Finally, their offspring hatches and grows on the surface of the host’s body, which is paralyzed but still alive, using it as a source of food. The mother stays with its offspring providing parental care [1,2,7,9,10].

Moreover, *S. domesticus* can be a sanitary injurious pest since it may accidentally sting humans who come near affected wooden objects [1-11]. Cases of injuries caused by the venom of *S. domesticus* and, to a much lesser extent, *Sclerodermus brevicornis* have been previously reported in Italy, including at least 25 affected men and 22 women with ages ranging from 18 to 74 years [1-6,8-12]. The exposure to worm-eaten furniture has definitely been identified as a risk factor for humans [1-12]. According to some authors, dermatitis caused by *S. domesticus* should be considered as an occupational disease for antique dealers and restorers [9-11]. In the last decade, a few cases of injuries caused by *S. domesticus* in humans have been documented in Italy [8-11]. Therefore, in order to draw attention to this bethylid wasp that causes medical problems, we report a case of *Sclerodermus* sp. stings in humans in this country.

## Case presentation

In mid-June 2013, a 40-year-old man brought a dead insect in a glass to our department for identification and orientation on the medical importance of its stings. The man reported that, in early June, he and his wife had moved from northern Italy to the province of Pisa (Tuscany, Central Italy), to spend a holiday in a seaside town. As soon as they settled in the rented furnished house, they were victims of painful burning stings. They occurred repeatedly, always inside the house, suddenly and in undetermined circumstances, several times during the day or evening, never at night while they were sleeping. The stings were inflicted on the dorsum, abdomen, arms, and thighs. On the sting sites, raised red itchy rash developed. During one of these episodes, the man hit his right thigh with the hand on the skin area where he felt the sting. After that, he looked at the palm of his hand and saw a crushed insect. He then decided to keep the insect and bring it in to be identified.

**Figure 1:**
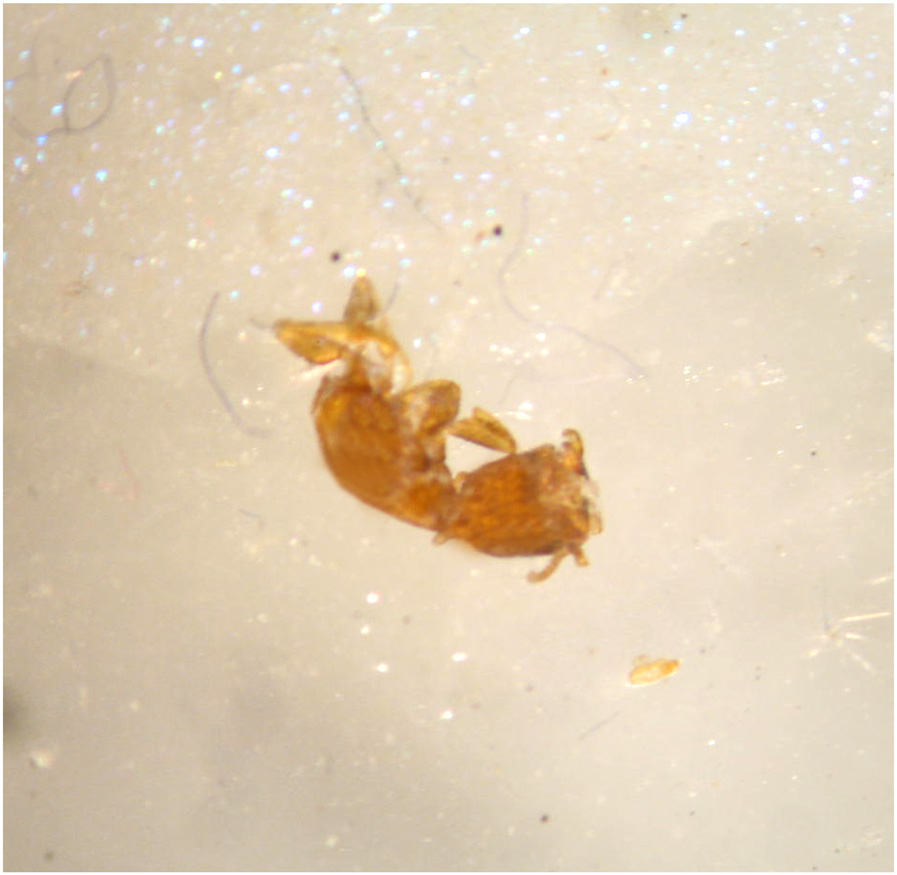
**Dorsal view of the cephalothorax of *Sclerodermus *sp. by stereoscope.** The head is visible while the antennae are only partially visible.

**Figure 2:**
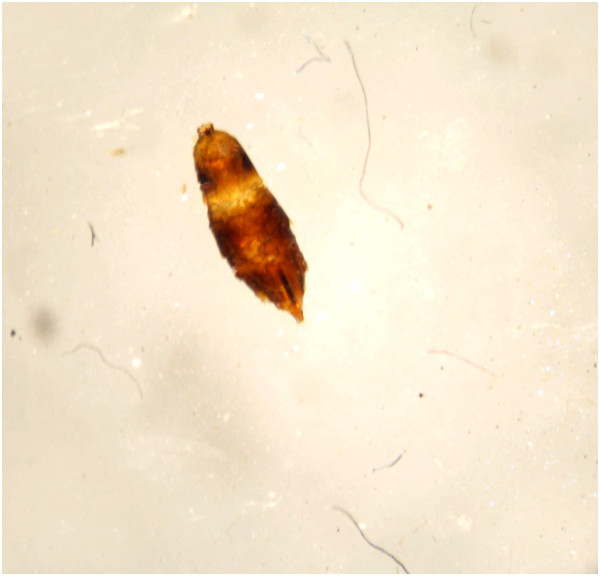
**Dorsal view of the abdomen of *Sclerodermus *sp. by stereoscope.** Abdominal segments are partially visible.

On arrival, the insect was examined by the author in relation to its morphological characteristics with the aid of a stereoscope (Zeiss Stemi DV4, Germany). The insect was very damaged and divided into two distinct parts (cephalothorax and abdomen). It was dark brownish in color and very similar to an ant (Figures 1 and 2). Since the morphological characteristics were consistent with those previously described and shown by other authors [2,4,6,7,9-11], the insect was identified as a specimen of *Sclerodermus* sp.

**Figure 3:**
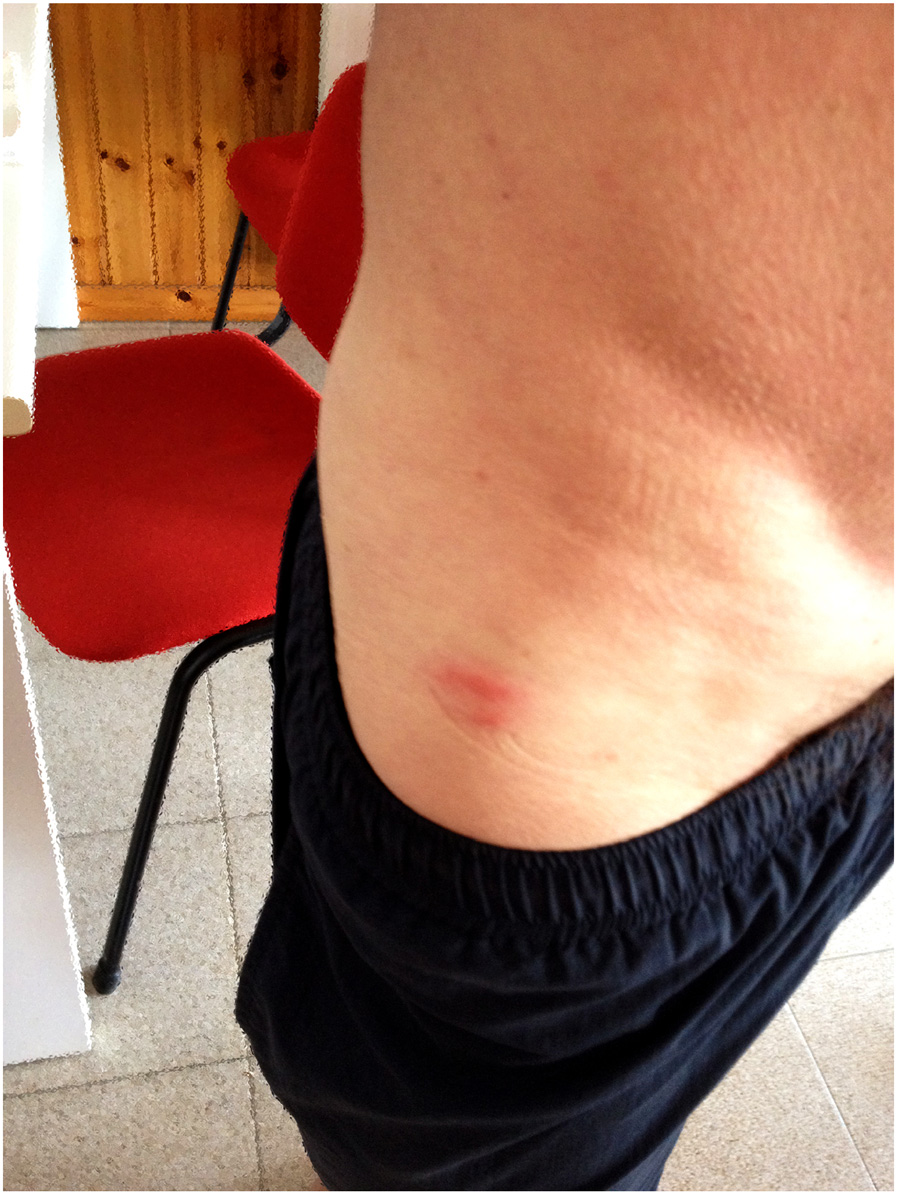
**Raised red itchy rash on the skin of the right thigh of a 40-year-old man stung by *Sclerodermus *** **sp.**

The next day, the man was contacted by phone and informed on the outcome of the morphological examination and on the biological characteristics of the insect. He expressed his willingness to gather information from the landlord and check the furniture. He also agreed to provide a photo (Figure 3). Two days later, the man reported that the house had always been furnished with second-hand furniture. In early May, a sofa had been purchased from a dealer of used furniture and placed in the living room. He and his wife had realized that this sofa was worm-eaten due to the presence of small amounts of wood dust on the floor and small holes in the wood. About ten days later, the man was contacted for the last time. He and his wife had returned home, had consulted their family doctor, and were pretty much healed after 3 to 4 days of treatment.

## Discussion

*S. domesticum* has a cosmopolitan distribution but may be found mainly in temperate climates [10]. Besides Italy, stings in humans by *Sclerodermus* species have been described in the Balkans, China, Congo, France, Greece, Japan, North Africa, Sierra Leone, Spain and Swiss [2,4,5,7]. In such cases, *S. domesticus*, *S. brevicornis*, *Sclerodermus nipponensis*, *Sclerodermus abdominalis* and *Sclerodermus unicolor* were involved [7,13]. Based on the literature, human injuries caused by species other than *S. domesticus* are very sporadic. Therefore, we believe that *S. domesticus* was the species most likely involved in our case. However, the lack of intact morphological characters in the only specimen examined did not allow the identification at species level and, thus, precluded any definitive conclusion.

Reports of injuries caused by stings of *S. domesticus* or *S. brevicaulis* generally indicate a single individual, but occasionally are observed in people sharing the same workplace or the same household [2,4,12], as in our case. Wood-eating larval stages of Anobiid beetles can be found whenever suitable wood products are available (homes, offices, garages, warehouses, attics, cellars). Since *S. domesticus* and *S. brevicaulis* females wander searching for their host larvae, they can be found whenever there are Anobiidae larvae and, thus, can be considered as common synanthropic insects. Probably, this is the reason why a large number of *Sclerodermus* stings in Italy were inflicted in humans indoor, mostly at home as in this case, but sometimes in the workplace [1-6,8-12].

Nonetheless, some cases of *S. domesticus* stings were associated with outdoor activities [5]. Probably, the females were searching for larval forms of Coleoptera or Lepidoptera in dead trees, fallen logs and branches, firewood, fences, and so on. *S. domesticus* stings in humans have been reported either during the day or at night, but especially at night [2-4,8]. Other reports showed that stings may occur under undefined circumstances [9-11]. However, there are records of people being stung under well identified occasions: sitting on an old chair or wearing clothes taken from a wardrobe, and even lying in bed since this bethylid wasp is also found in wool mattresses [2,4,6,10,11].

In our case, a worm-eaten sofa was identified as source of infestation with *Scleroderma* sp. Probably, the bethylid wasps climbed on their clothes while the man and his wife were sitting on the sofa, during the day and evening. Then, later, the insects stung at any time, as soon as they were able to penetrate through clothes and to come into contact with the skin. Before going to sleep the man and his wife took their clothes off, leaving them far away from the bed. In addition, the living room with the worm-eaten sofa was away from the bedroom. Therefore, probably for these reasons, the man and his wife were not exposed to risk of stings by *Sclerodermus* sp. during the night.

It is likely that fabric can serve as fomite for *Sclerodermus*. In other investigations, it was observed that the stings occurred mostly in areas of the body covered by clothing or especially when clothes were taken from an old worm-eaten wardrobe [2,4]. In Spain, the presence of *S. domesticus* in houses was curiously associated with new curtains (probably sewn and purchased in a contaminated shop) in the living room and with a pile of old newspapers [14]. It has also been reported that stings by the mite *Pyemotes ventricosus*, an ectoparasite of the larvae of Anobiid beetles in wood furniture, may be a cause of human dermatitis following exposure to worm-eaten furniture [15]. *P. ventricosus* and *S. domesticus* can be found in the same environments [5]. Since environmental investigations were not carried out, the possible co-occurrence of stings by *P. ventricosus* and by *S. domesticus* cannot be ruled out in this study.

Multiple *S. domesticus* stings (up to 40 in a subject) and for prolonged periods of time (several weeks or months) have previously been documented, including the recurrence of continued seasonal episodes over several years in spring [5,9]. Stings took place either in urban or in rural areas from February to October [2,4]. In agreement with the present findings, however, they were more common in urban environments during spring and summer [2,4,5]. Previously reported body parts affected by *S. domesticus* stings included not only regions of the arms, legs, and trunk – as described in this case – but also the neck, hands, and pelvis [2-6,8-11].

Studies on hymenopteran venoms are commonly related to honey bees (*Apis mellifera*), wasps of the family Vespidae and stinging ants, including some species of the genera *Solenopsis*, *Pachycondyla*, and *Myrmecia*[16-19]. Although *S. domesticus* and other related species may be medically important insects, their venoms have not drawn much attention of the scientific community. It can be speculated that the composition of *S. domesticus* venom is similar to that of venoms of other Hymenoptera, mostly Bethylidae [7]. Some studies have shown that Hymenoptera venoms are composed of a mixture of several proteins, peptides, toxins, enzymes, biogenic amines, and allergens [16,17]. The risk of sensitization in humans increases with age [16]. It is known that insect venom composition may vary remarkably and differences in components (both presence and abundance of individual compounds) can be found at all taxonomic levels (family, genus, species) [7]. Therefore, detailed composition and possible mechanisms of action of *S. domesticus* venom still remain unclear.

The most typical symptom of a *Sclerodermus* sting is an immediate, intense and persistent, painful sensation, sometimes associated with burning sensation [1,2,4,5,8-10]. Only in one reported case the sting was unnoticed and local skin reactions were mild [2]. In some observations, stings were shortly followed by the appearance of erythematous purple dome-shaped papules, measuring 0.5-1 cm in diameter [1,2,4,9-11]. In other reports there are descriptions of vesicles with purulent material or wheals that joined together to form larger urticarial patches [2,4,5,8,12]. Skin lesions are frequently associated with an intense itching sensation [1,2,4,9-12].

The onset of general symptoms and signs generally includes general discomfort, general malaise and fever, or malaise associated with fever, nausea, and dizziness requiring hospitalization [2,4,9,10]. Other reported effects are related to anaphylaxis, including generalized or local edema, chest tightness, abdominal pain, vomit, cyanosis, dysphagia, hoarseness, sense of confusion, shock status, lower blood pressure, urinary and fecal incontinence, and loss of consciousness [1]. Laboratory findings showed that numerous erythematous-hemorrhagic papules and fever were associated with slight increasing of fibrinogen and glycemia [6]. Histopathological examination is usually characterized by edema and vasodilatation in superficial and mid-dermis. Furthermore, a predominantly perivascular inflammatory infiltrate consisting of neutrophils and lymphocytes, with some eosinophils, has also been observed [10,11].

If the manifestations are local, prognosis is good and lesions can disappear spontaneously within 3 to 14 days [2,4,14]. Insect sting anaphylaxis can be very serious and have a dramatic follow-up, unless the patient is immediately treated. Most cases require epinephrine, oxygen supply and intensive care procedures provided to the patient [20]. If control measures against *Sclerodermus* are not taken, symptoms are not relieved by common treatment with systemic anti-histaminic, topical corticosteroid and antiparasitic drugs [9]. When environmental pest control is carried out, fever disappears within 3 days and skin lesions ameliorate in 7 to 10 days without therapy [6,7,9]. Environmental control measures comprise the complete removal of worm-eaten furniture or their treatment with proper products [4]. Complete remission with prevention of a periodic recurrence of symptoms can be achieved by means of symptomatic treatment (e.g. topical corticosteroids and oral antihistamines) in patients, associated with furniture pest control by using synthetic pyrethroids such as permethrin, deltamethrin, and 0.1% cyfluthrin [8,10,11].

## Conclusion

Our findings corroborate previous reports in Italy. In case of skin lesions caused by *Sclerodermus*, an etiologic diagnosis may be difficult. Pain followed by swelling, urticarial wheals or papular urticaria, and itch comprise a common clinical picture in cases of insect stings and other arthropod bites [21-23]. The possible presence of systemic symptoms such as fever and general malaise may also occur in these cases [24,25]. Based on an accurate history, including the possible exposure to worm-eaten furniture at home or in the workplace, *Sclerodermus* stings can be suspected. However, a definitive diagnosis requires a *Sclerodermus* specimen collected during or after the sting and the identification by an expert. These conditions rarely occur due to the short term activity of *Sclerodermus* on human skin. The direct examination of house dust has been proposed as a valid diagnostic method to identify the presence of *Sclerodermus* and other insects or mites of medical interest in houses [3,5]. However, this technique requires specific skills and well trained personnel. For these reasons, it is possible that many cases of *Sclerodermus* stings are misdiagnosed and, thus, their incidence is underestimated [2,4]. To avoid long-term and ineffective therapies or expensive and unnecessary diagnostic procedures, the present report is a reminder that adequate knowledge concerning epidemiology, clinical signs, diagnosis, treatment and control of *Sclerodermus* stings should be required from physicians, dermatologists, medical and public health entomologists as well as to common people (mostly specific categories of workers) who are at risk of exposure to worm-eaten furniture [9].

## Consent

Written informed consent was obtained from the patient for publication of this case report.

## Competing interests

The author declares that there are no competing interests.
